# A novel hypoxic lncRNA, HRL-SC, promotes the proliferation and migration of human dental pulp stem cells through the PI3K/AKT signaling pathway

**DOI:** 10.1186/s13287-022-02970-5

**Published:** 2022-06-28

**Authors:** Junkai Zeng, Ming Chen, Yeqing Yang, Buling Wu

**Affiliations:** 1 Nanfang Hospital, Southern Medical University, Guangzhou, People’s Republic of China; 2 Shenzhen Stomatology Hospital (Pingshan), Southern Medical University, Shenzhen, 510515 Guangdong People’s Republic of China; 3 Stomatological Hospital, Southern Medical University, Guangzhou, People’s Republic of China; 4 School of Stomatology, Southern Medical University, Guangzhou, People’s Republic of China

**Keywords:** Human dental pulp stem cells, Hypoxia, lncRNA, Proliferation and migration

## Abstract

**Background:**

Human dental pulp stem cells (hDPSCs) are critical for pulp generation. hDPSCs proliferate faster under hypoxia, but the mechanism by which long noncoding RNA (lncRNA) regulates this process is not fully understood.

**Methods:**

Novel lncRNAs were obtained by reanalysis of transcriptome datasets from RNA-Seq under hypoxia compared with normoxia, and a differential expression analysis of target genes was performed. Bioinformatics analyses, including gene ontology analysis, Kyoto Encyclopedia of Genes and Genomes pathway analysis and gene set enrichment analysis, were used to understand the function of key novel lncRNAs. hDPSCs were isolated from dental pulp tissue. EdU and scratch wound healing assays were used to detect the proliferation and migration of hDPSCs. qRT-PCR was used to detect changes in the RNA expression of selected genes. RNA fluorescence in situ hybridization, small interfering RNA, qRT-PCR and Western blot analysis were used to explore the function of key novel lncRNAs.

**Results:**

We identified 496 novel lncRNAs in hDPSCs under hypoxia, including 45 differentially expressed novel lncRNAs. Of these, we focused on a key novel lncRNA, which we designated HRL-SC (hypoxia-responsive lncRNA in stem cells). Functional annotation revealed that HRL-SC was associated with hypoxic conditions and the PI3K/AKT signaling pathway. HRL-SC was mainly located in the cytoplasm of hDPSCs and had stable high expression under hypoxia. Knockdown of HRL-SC inhibited the proliferation and migration of hDPSCs and the expression levels of PI3K/AKT-related marker proteins. Furthermore, the AKT activator SC79 partially offset the inhibitory effect caused by the knockdown, indicating that HRL-SC promoted hDPSCs through the PI3K/AKT signaling pathway.

**Conclusions:**

Hypoxia-responsive lncRNA HRL-SC promotes the proliferation and migration of hDPSCs through the PI3K/AKT signaling pathway, and this understanding may facilitate the regenerative application of hDPSCs.

**Supplementary Information:**

The online version contains supplementary material available at 10.1186/s13287-022-02970-5.

## Introduction

Human dental pulp stem cells (hDPSCs) are located in dental pulp tissue and have a high capacity for self-renewal and multidirectional differentiation [1]. Dental pulp tissue can be obtained from discarded human teeth, including wisdom teeth or premolars extracted for orthodontic reasons [2]. The advantages of hDPSCs include little ethical controversy, low immunogenicity and strong proliferation ability compared with bone marrow mesenchymal stem cells (BMSCs) [3, 4]. In addition, hDPSCs can differentiate into primary odontoblasts and pulp cells to form pulp-like tissues. Thus, hDPSCs are ideal seed cells for dental pulp tissue engineering and have been widely used in pulp regeneration research [5, 6].

R. Schofield first proposed the concept of stem cell niches, which refers to a specific microenvironment that regulates the fate of stem cells [7]. Stem cells in vivo reside in a hypoxic microenvironment, which is one of the most important stem cell niches that maintains stem cell characteristics [8]. The oxygen concentration of different hypoxic microenvironments depends on the organ blood flow; thus, less irrigated organs receive less oxygen, ranging from 1 to 14% [9, 10]. The oxygen concentration of mesenchymal stem cells in bone marrow has been reported to be 1–7% [11]. Dental pulp tissues are surrounded by rigid dentin, and the apical foramen is the only vascular access, indicating that the pulp tissues are also at lower oxygen tension levels [12]. It has been reported that the oxygen concentration of rat incisor pulp is approximately 3% [12]. In general, hDPSCs are cultured in 21% oxygen under in vitro conditions, and high oxygen tension increases oxidative stress, resulting in damage to cell metabolism [13]. Therefore, hypoxic culture provides a better simulation of the physiological conditions that are necessary for stem cell culture [14]. Hypoxia enhances the proliferation and migration of hDPSCs [15–17]. In addition, increased expression of pluripotency markers in hDPSCs such as SRY-box transcription factor 2 (SOX2), octamer-binding protein 4 (Oct-4) and c-Myc were observed under hypoxia [18]. The hypoxic signaling response is orchestrated by hypoxia inducible factor (HIF), which activates glycolytic enzymes to provide energy for stem cells [19, 20]. Furthermore, because hypoxia maintains an undifferentiated state and promotes the proliferation of stem cells, it is necessary to explore the mechanisms of stem cell behavior under hypoxia.

lncRNAs are a class of RNA molecules of more than 200 nt without protein coding potential [21]. lncRNAs are involved in stem cell regulation at the transcriptional, posttranscriptional and epigenetic levels, interacting with molecules to act as guides, scaffolds, decoys and tethers to regulate cell proliferation, migration, apoptosis and differentiation [22–24]. There is a group of lncRNAs that is specifically expressed under hypoxia, called hypoxia-responsive lncRNAs (HRLs) [25, 26]. Increasing evidence indicates that lncRNAs participate in the hypoxia-related cellular processes of stem cells, including proliferation, migration, differentiation and stemness. Some studies have demonstrated that lncRNAs that are modulated by hypoxia promote the proliferative and migratory capacity of mesenchymal stem cells [27–29]. For example, lncRNA-miRNA–mRNA network analysis with lncRNAs as the core components was performed to understand the molecular expression patterns of hypoxia-promoting effects in olfactory mucosa mesenchymal stem cells [27]. It was also revealed that the hypoxia‐responsive lncRNA STL may be the key component of a coding-noncoding gene coexpression network that regulates the functions of stem cells [30].

However, very few studies have explored the roles of lncRNAs in the regulation of hDPSCs, and the mechanisms involved remain largely unclear. Here, we reanalyzed our previous RNA-seq data of hDPSCs under hypoxia and normoxia, and performed a differential expression analysis of target genes to determine the functions of novel lncRNAs. We identified a key novel lncRNA, HRL-SC, which was significantly upregulated in hDPSCs under hypoxia compared with normoxia. Functional annotations revealed that HRL-SCs were closely associated with hypoxia signatures. Knockdown of HRL-SC inhibited the proliferation and migration of hDPSCs under hypoxia and decreased the activity of the PI3K/AKT signaling pathway. However, the activators of AKT offset the inhibition caused by knockdown of HRL-SC. Our data show that hypoxia-responsive HRL-SC promotes the proliferation and migration of hDPSCs via the PI3K/AKT signaling pathway and provides a potential molecular mechanism of hDPSCs under hypoxia.

## Materials and methods

### RNA-Seq data processing

RNA-Seq had been performed for hDPSCs under normoxia and hypoxia in our previous study, which can be obtained from NCBI GEO: GSE118046. Fastqc (version 0.11.9) was used to access the sequencing quality and Trimmomatic was used to filter low-quality sequencing reads. After the quality control, reads were aligned to the reference genome (Hg19) using HISAT2 (version 2.1.0) and the sam files for each sample were obtained. Next, sam files were converted to sorted bam files using Samtools (version 1.13). Assembly of bam files and gene quantification (FPKM values) were using StringTie (version 2.1.5) and the '-merge' function was performed to merge the transcript.

### Identification of novel lncRNA

The pipeline for identifying novel lncRNA from assembled transcript are as follows: (1) filter transcripts with class code ‘i, j, u, x’ from Gffcompare of StringTie; (2) remove the transcripts that matched annotations of known lncRNA; (3) filter transcripts with length ≥ 200 bp, exon numbers ≥ 1; (4) filter transcripts without coding potential using the protein-coding gene prediction tools including Coding-Non-Coding-Index (CNCI, version 2), Coding Potential Calculator (CPC, version 2), Coding Potential Assessment Tool (CPAT) and Pfam-scan (version 14.3) [31]. Finally, retaining transcripts with FPKM > 0 in at least one sample were novel lncRNAs.

### Differential expression analysis

Differential expression analysis was used to identify the key novel lncRNAs under hypoxia. Expression matrix of FPKM values for mRNA and novel lncRNA was imported into R (version 3.63) and were analyzed using Linear Models for Microarray data (limma, https://bioconductor.org/packages/limma/) package in Bioconductor. Up- or downregulated differentially expressed mRNA/novel lncRNAs between samples under hypoxia and normoxia were identified with the cutoff criteria of *P* < 0.05 and |fold change (FC)|> 2.

### LncRNA target prediction and functional annotation analysis

To understand the function of key lncRNA, LncTar (version) was used to explore the potential interaction between key lncRNA and differentially expressed genes. LncTar is a bioinformatics tool that calculates the free energy between lncRNA and target genes and a threshold of -0.1 ndG (default parameter) was set. Next, the target genes were imported into Database for Annotation Visualization and Integrated Discovery (DAVID) (https://david.ncifcrf.gov/) to perform GO enrichment and KEGG pathway analysis. To further explore the function of lncRNA HRL-SC, GSEA (version 4.1.0) was performed using c2.all.v7.4.symbols.gmt (http://software.broadinstitute.org/gsea/downloads.jsp) as a reference gene set. *P* < 0.05 and FDR < 0.25 were considered statistically significant.

### Cell culture and identification

The hDPSCs were obtained from the third molar of patients aged 18–25 years indicated for extraction at the Department of Stomatology in Nanfang Hospital, Southern Medical University, Guangzhou, Guangdong, China. All experimental protocols were approved by the Ethical Committee of Southern Medical University. The isolated hDPSCs were cultured in Dulbecco modified eagle’s medium (DMEM) supplemented with 10% fetal bovine serum (FBS; ExCell Bio, Shanghai, China), 100 U/mL penicillin and 100 μg/mL streptomycin (Sigma, St. Louis, Mo, USA) at 37 °C in humidified atmosphere containing 5% CO_2_ with different oxygen concentrations. Induced conditioned medium was used for cell induction and differentiation. The medium was replaced with the medium containing 5 µg/mL of SC79 (10 μM, GlpBio) in AKT activation experiments. The culture media were renewed every 3 days.

After 21 days of differentiation, Alizarin Red and ALP activity staining were performed to verify the formation of mineral nodes and purple-colored precipitate. Besides, the cells were incubated for surface markers including CD45, CD34, CD90, CD44 and CD29 and then were detected by flow cytometry to identify the cell phenotypes of hDPSCs.

### Cell transfection

The siRNAs against lncRNA and control siRNA were obtained from GeneChem (Shanghai, China). The details of sequences could be seen in Table 1. The siRNAs and control siRNA were transfected using riboFECT™ CP (Ribobio, Guangzhou, China) according to the manufacturer's protocol.

**Table 1:** Primers for the qRT-PCR and probes used in cell transfection

Gene name	sense (5′–3′)	Anti-sense(5′–3′)
β-actin	TCACCATGGATGATGATATCGC	ATAGGAATCCTTCTGACCCATGC
MSTRG.20649.1	TGGAGGGAAGACACTGTCCTAT	TGCTTGGTGGGGGTTATCCA
MSTRG.8144.1	GGATTGTTCCCCACAAACCA	ATTCCAGTAACCTGCCCTTGG
MSTRG.27918.3	TCAAAGTGGGTCAGTGCAGG	CCTCAGGCCAACAGAGTAGC
MSTRG.31275.1	TCTCACATCCAATTCCCCGT	GAGTGCCACCTAGTGCTACC
MSTRG.16037.1	GAAAAGGGGTGTCAGCAACAA	TCACACAGACATCACTCCGTC
MSTRG.6909.1	AGCTGCAAAAGGCATCCTAGT	CCCCACAGCGTTTAATGGAAG
MSTRG.11228.1	TCGGGTGATGATGGTTCCAC	GAACCACCTCCCGAACCCAA
MSTRG.10485.1	CGGTGGCTCACTCATTGACA	TGGACTTGGTCTGTTAAGGGC
MSTRG.15583.1	TGCCACATCGTGTGTTCTGT	AGACACGGTAACCACACACC
MSTRG.11101.4	CAGACGCTCCCCGCAGA	ATCAAGACACCTGCCTTCCA
PDK1	CTGTGATACGGATCAGAAACCG	TCCACCAAACAATAAAGAGTGCT
siRNA1	CCACAAAUGGCCUGAACUGUATT	UACAGUUCAGGCCAUUUGUGGTT
siRNA2	CCUCAGGUAGCUGCUGUUUGUTT	ACAAACAGCAGCUACCUGAGGTT
siRNA3	UGCUGUUUGUGUGAGAAAGAATT	UUCUUUCUCACACAAACAGCATT

### RNA-fluorescent in situ hybridization (RNA-FISH)

The subcellular localization of HRL-SC was detected by RNA-FISH kit (GenePharma, China) according to manufacturer’s protocol. Briefly, the treated hDPSCs were washed with PBS and fixed with 4% Paraformaldehyde for 15 min. Then, the cells were permeabilized with 0.5% Triton X-100 in PBS hybridization buffer and washed with SSC solution. Cy3-labeled HRL-SC probes was used to perform hybridization in the dark overnight. After being washed with 0.1% Tween 20 and SSC solution, the cells then were observed and photographed under an inverted fluorescent microscope.

### Scratch wound healing assay

The migration capability of hDPSCs was characterized by scratch wound healing assay. Cells were cultured with DMEM supplement with 10% FBS in 6-well plates until they reached confluence. After starving for 24 h with serum-free DMEM, scratches were generated with a 1-mL pipette tip in each well. After scratching for 0 h (right after scratch), 12 h and 24 h, the migration of cells was observed and photographed under an inverted microscope. The scratch wound healing area across each scratch was analyzed calculated by using ImageJ software.

### Cell proliferation assay

The proliferation capability of hDPSCs was measured using the EdU (meilunbio, Dalian, China) assay. The hDPSCs were labeled with EdU for 2 h and then fixed, washed, permeabilized and stained according to the manufacturer's protocol. After cells were washed, the DNA was stained with Hoechst 33342; then, each well was observed and photographed under an inverted fluorescent microscope. The quantification was performed using with analyze particles function of ImageJ software to count the number of positive cells. The proliferation rate was defined as the ratio of EdU-positive cells to total Hoechst-positive cells from at least three random fields of view.

### RNA extraction and RT-PCR

Total RNA was extracted from each group using EZ-press RNA Purification Kit (EZBiosicence, USA) according to the manufacturer’s protocol and quantified by NanoDrop ND-1000 (Thermo Scientific, USA).

Reverse transcription was performed using Color Reverse Transcription Kit (EZBiosicence, USA) with gDNA remover. A volume of 13 µL RNA solution of each sample (containing 1 µg RNA) was treated with 2 µL provided gDNA remover to remove genomic DNA. Each reaction was set for 20 µL, including 13 µL processed RNA solution and 5 µL 4× RT Master Mix.

### qRT-PCR validation

Real-time PCR was performed with Color SYBR Green qPCR Master Mix ROX2 plus (EZBiosicence, USA) on Roche LightCycler®480. Each reaction was set for 10 µL: 5 µL 2× Color Green qPCR Master Mix, 0.2 µL Forward Primer, 0.2 µL Reverse Primer, 3.6 µL RNase Free dH_2_O and 1 µL Product from RT reaction. All experiments were repeated three times, and relative gene expression was calculated using the 2-ΔΔCt method. The sequences of gene-specific primers are listed in Table 1.

### Western blot analysis

Cells of each group were lysed using the RIPA lysis buffer with protease inhibitors and the total protein contents were quantitated by the BCA assay (Beyotime, Shanghai, China). The samples were separated by 8% SDS-PAGE and then were transferred onto polyvinylidene difluoride (PVDF) membranes. The PVDF membranes were blocked in protein-free rapid blocking buffer (Epizyme Biomedical Technology, Shanghai, China) for 15 min and then incubated overnight at 4 °C with primary antibodies against β-actin, AKT, p-AKT, PI3K, p-PI3K (Bioss, Beijing, China), which diluted at 1: 1000. Then, the PVDF membranes were incubated with corresponding secondary antibodies (Proteintech, China). The bands were detected using ECL reagents (Biosharp, China). The densities of protein bands were quantified by Image J software.

## Results

### hDPSCs exhibit higher proliferation capacity under hypoxia

**Fig. 1:**
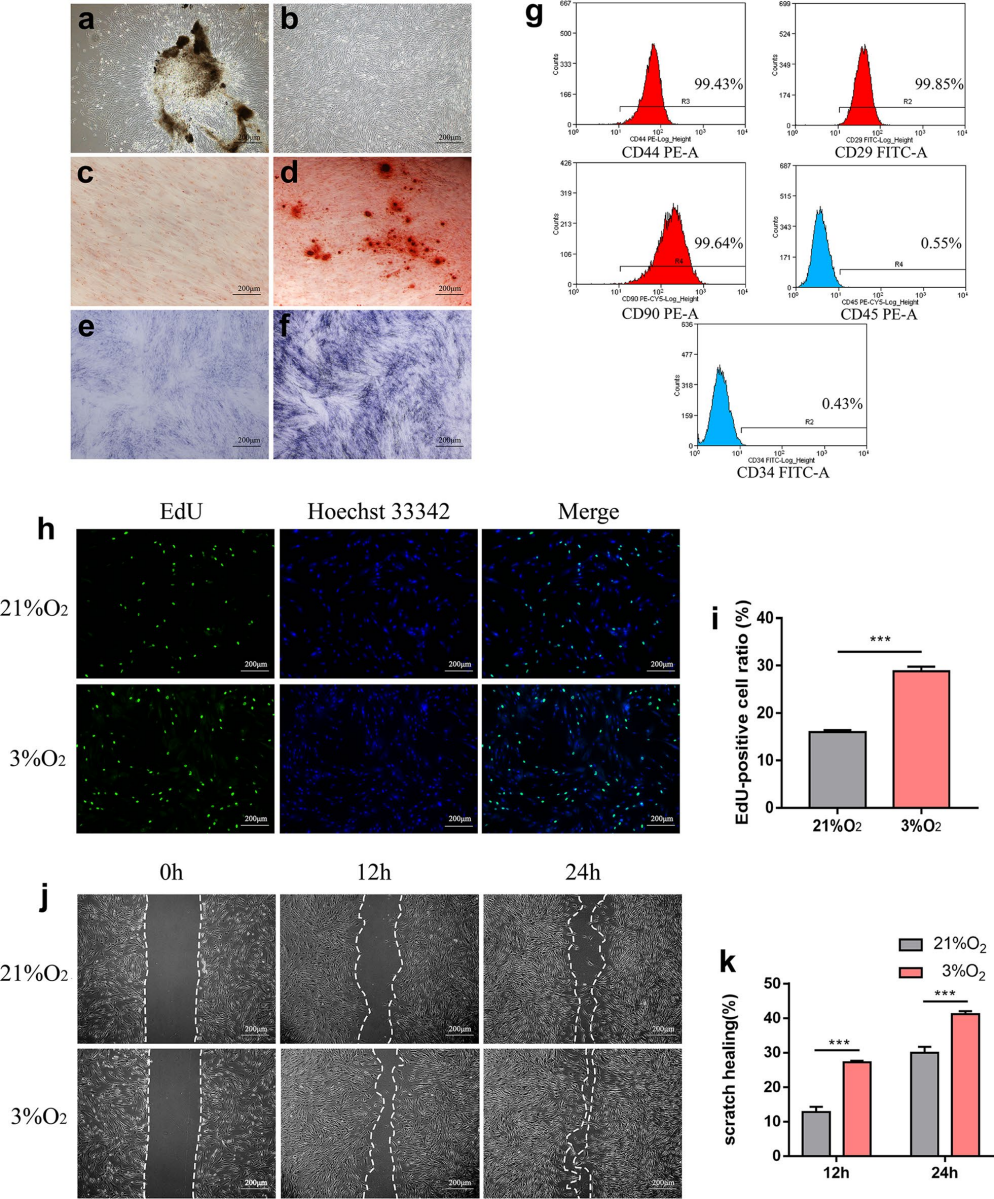
Identification of hDPSCs and promotion of hypoxia on hDPSCs. **a**, **b** Primary cultured and passage hDPSCs. Alizarin Red and ALP activity staining was performed and mineral nodes (**c**) and purple-colored precipitate (**d**) were detected in the induced group but not appeared in the non-induced group (**e**, **f**). **g** Surface markers of hDPSCs were detected by flow cytometric analysis, hDPSCs were positive for CD44, CD29, CD90 and negative for CD45, CD34. **h** EdU assay of cell proliferation. EdU-positive cells were in red and cell nuclei were dyed in blue. **i** EdU-positive cell ratio in normoxia group vs. hypoxia group. **j** Migration capability was characterized by scratch wound healing assay. **k** Scratch wound healing area in normoxia group vs. hypoxia group. ***P < 0.001

Primary hDPSCs were successfully isolated from dental pulp tissue and passaged (Fig. 1a, b). The results of staining showed that hDPSCs processed differential capacity (Fig. 1c–f). Flow cytometry results showed that hDPSCs were positive for stem cell markers CD44, CD29 and CD90 but negative for hematopoietic cell markers including CD45 and CD34 (Fig. 1g). Then, the migration and proliferation were, respectively, detected by scratch wound healing assay and EdU assay. As indicated by Fig. 1h–k, hDPSCs proliferated and migrated faster under hypoxia compared with normoxia.

### Identification and characterization of novel lncRNA in hDPSCs under hypoxia

**Fig. 2:**
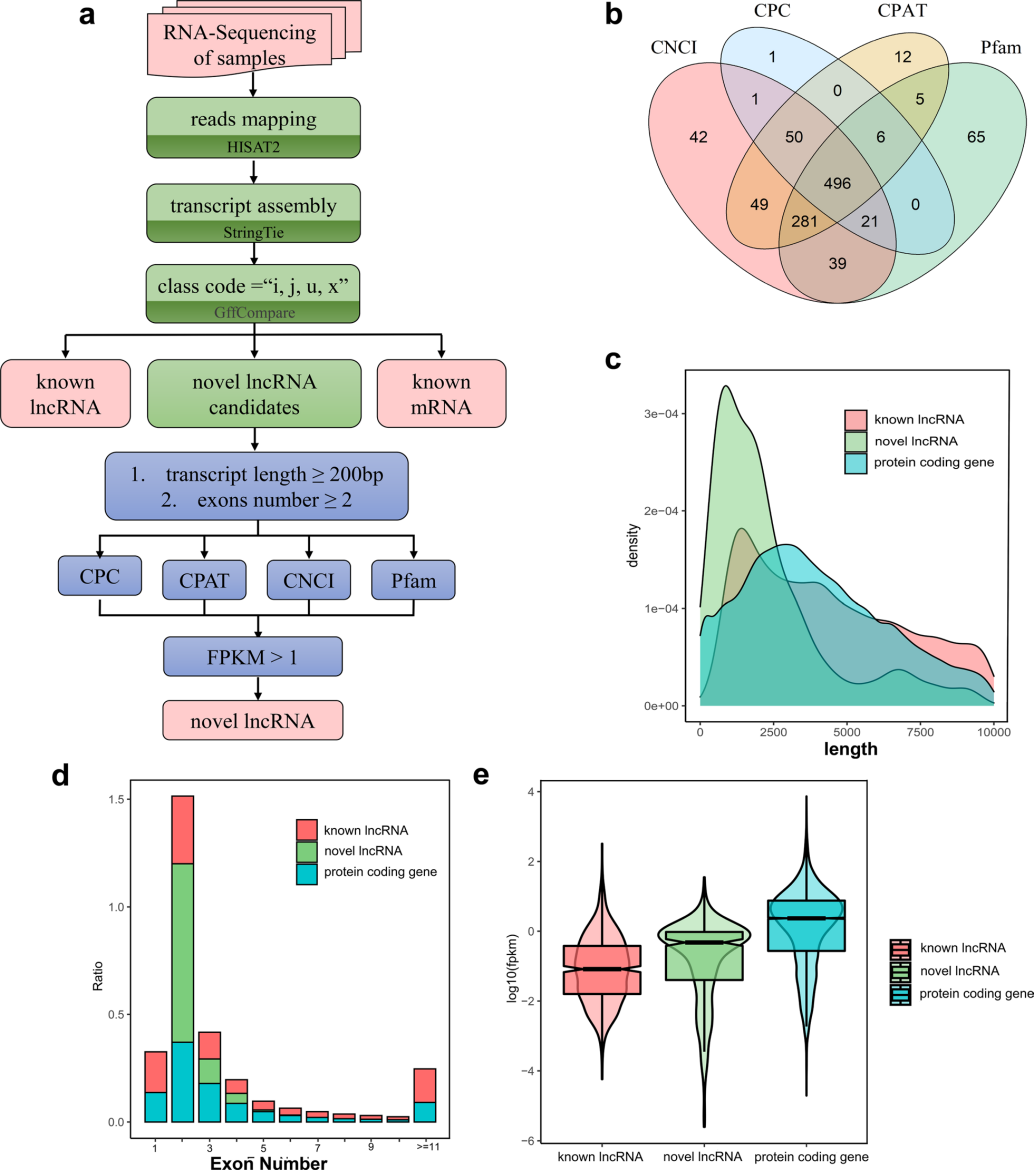
Identification and characterization of novel lncRNAs in hDPSCs under hypoxia. **a** Pipeline for novel lncRNAs identification. **b** Venn diagram showed the intersection of protein-coding potential analysis by CNCI, CPC, CPAT and Pfam-scan. **c** Transcript length distribution of known lncRNA, novel lncRNA and protein coding gene. **d** Distribution of exon number in known lncRNA, novel lncRNA and protein coding gene. **e** Expression level of known lncRNA, novel lncRNA and protein coding gene

To identify the lncRNAs involved in the hypoxic process, the RNA-Seq data was reanalyzed. According to pipeline (Fig. 2a), novel lncRNAs that may play an important role in regulating hDPSCs under hypoxia were obtained. Compared to the references in GENCODE database, 16,498 protein coding genes, 1351 known lncRNAs were obtained. According to the exon number and transcript length, we further filtered and acquired 1600 potential novel lncRNA transcripts. Then, the coding potential of transcripts was analyzed by CPC, CNCI, CPAT and Pfam-scan, and finally 496 novel lncRNAs that met the definition of lncRNA were obtained (Fig. 2b). Briefly, the novel lncRNAs show longer transcript length, fewer exons, lower coding potential and lower FPKM values, consistent with the characterization of lncRNA as previously reported (Fig. 2c–e)[32].

### Differential expression analysis and validation of novel lncRNAs

**Fig. 3:**
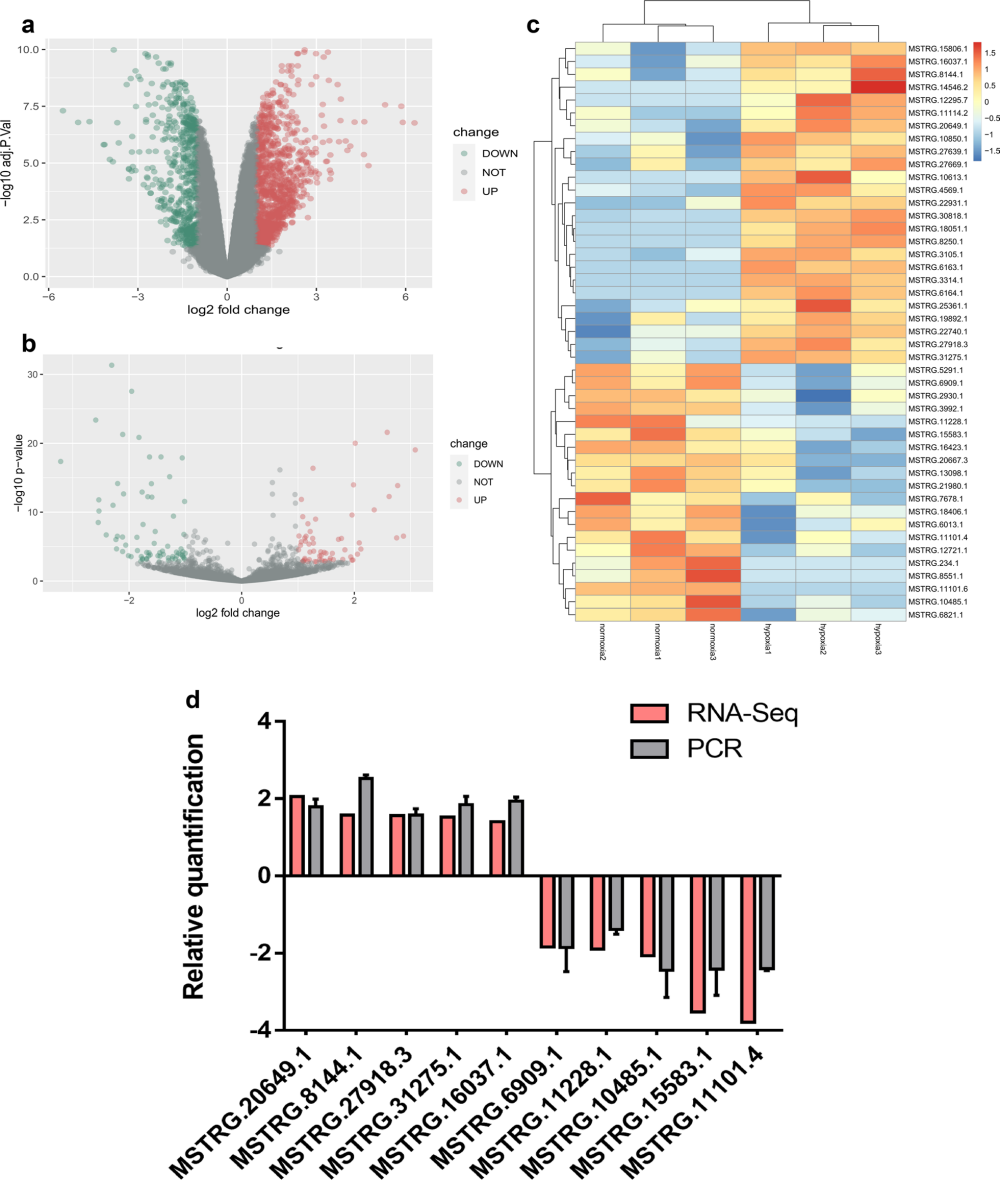
Differential expression analysis between normoxia and hypoxia group and expression level validation. **a** Volcano plot of differentially expressed genes, including 1123 upregulated genes and 561 downregulated genes under hypoxia. **b** Volcano plot of differentially expressed known lncRNAs, including 60 upregulated lncRNAs and 1123 downregulated lncRNAs under hypoxia. **c** Heatmap of differentially expressed novel lncRNAs. **d** The expression level of novel lncRNAs in RNA-seq data was validated by qRT-PCR

A total of 1684 differentially expressed genes (DEGs), 135 differentially expressed known lncRNAs and 45 differentially expressed novel lncRNAs (DENLs) were screened under hypoxia compared with normoxia, shown in the volcano plot and heatmap (Fig. 3a–c). Details of the differential expression analyses are provided in Additional file 2. The differential expression of novel lncRNAs was validated by RT-qPCR. The expression levels of novel lncRNAs calculated from qRT-PCR results were similar to those from RNA-Seq data (Fig. 3d). Among them, MSTRG.20649.1 was stably upregulated under hypoxia compared with normoxia. Therefore, we treated MSTRG.20649.1 as the key novel lncRNA that may regulate proliferation and migration of hDPSCs under hypoxia, designated it as lncRNA HRL-SC (hypoxia-responsive lncRNA in stem cells).

### Identification, location and expression pattern analysis of lncRNA HRL-SC

**Fig. 4:**
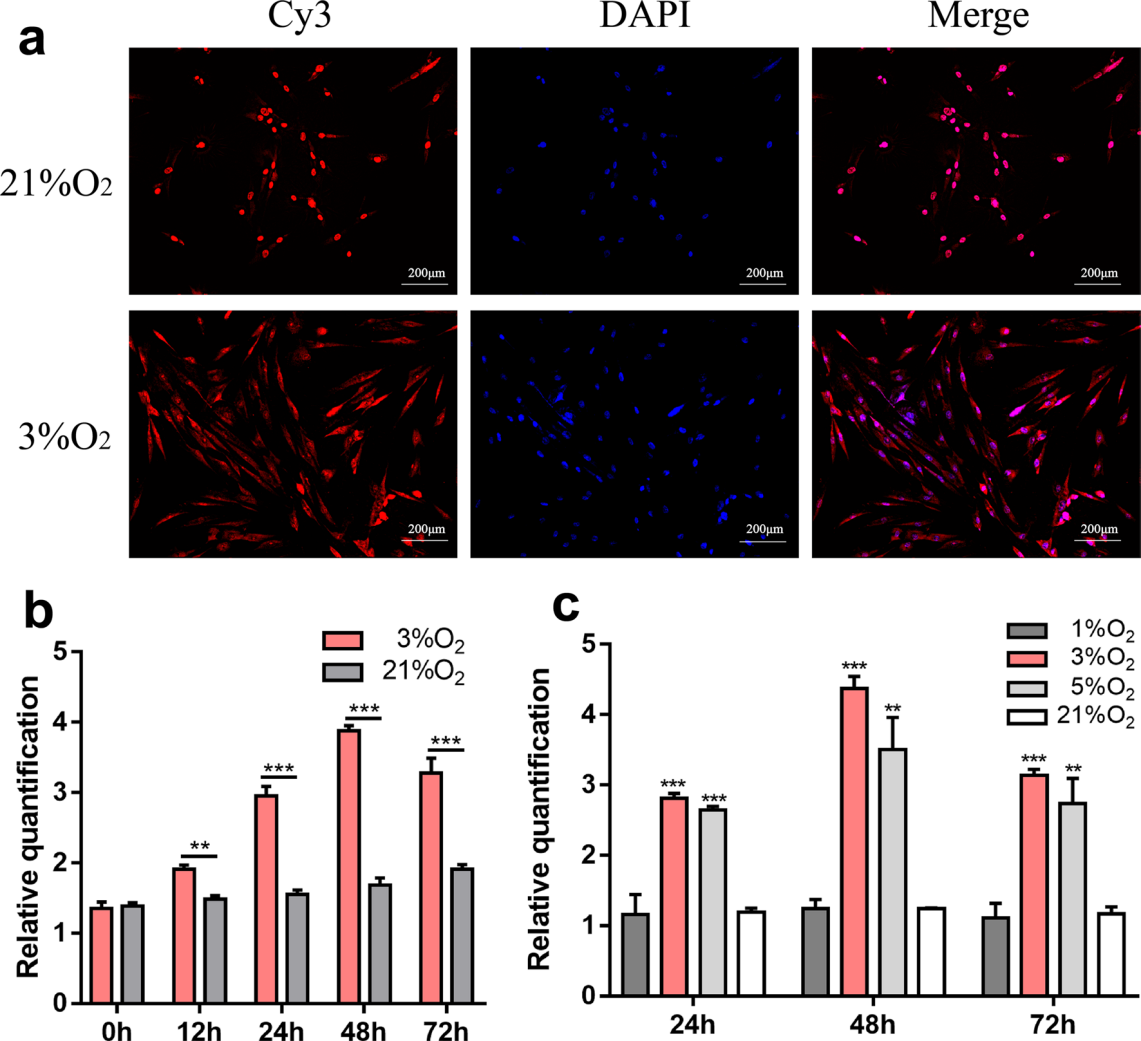
Location and expression pattern analysis of lncRNA HRL-SC. **a** The subcellular localization of HRL-SC examined by RNA-FISH test under normoxia and hypoxia. The FISH probes against HRL-SC were dyed in red by Cy3 and the cell nucleus were dyed in blue by DAPI. **b** Expression levels of HRL-SC under hypoxia with different exposure durations using qRT-PCR. **c** Expression levels of HRL-SC under different oxygen concentrations using qRT-PCR

lncRNA HRL-SC is an intronic lncRNA and located in chromosome 3, from locus 48,566,603 to locus 48,567,458 (Additional file 1: Fig. 1a). RNA-FISH test was performed to examine the subcellular location of HRL-SC, which showed that HRL-SC was mainly located in the cytoplasm of hDPSCs under hypoxia but in the nucleus under normoxia (Fig. 4a). To further understand the expression pattern of HRL-SC in hDPSCs, we detected its expression levels under different oxygen concentrations with different exposure durations. The RT-qPCR results showed that HRL-SC had a higher expression level in 3%O_2_ when compared with 1%O_2_, 5%O_2_ and 21%O_2_ (Fig. 4b). In addition, the dynamic expression levels of HRL-SC were also observed in hDPSCs under hypoxia (Fig. 4c).

### Target genes and pathway analysis of lncRNA HRL-SC

**Fig. 5:**
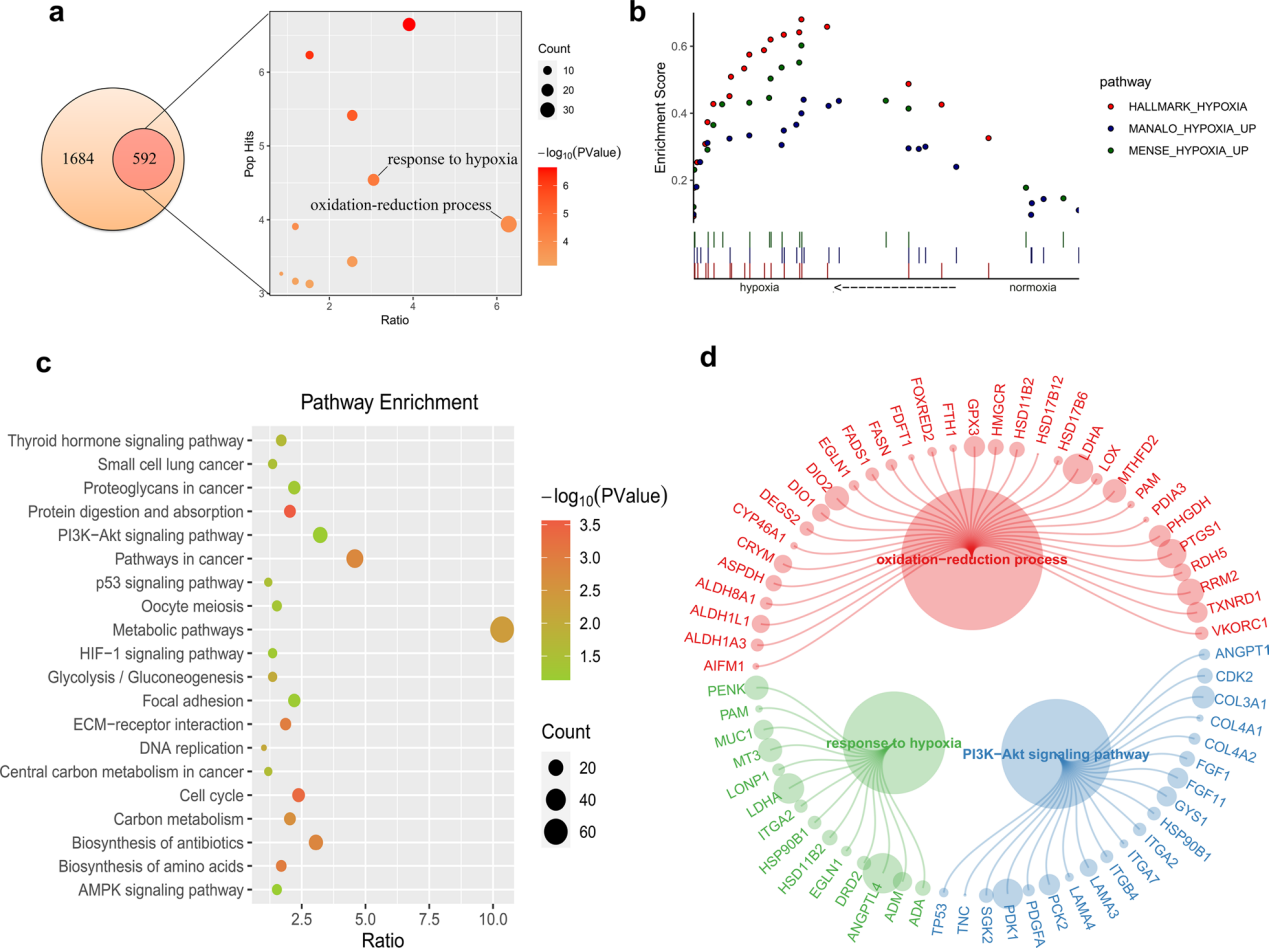
Functional annotation of lncRNA HRL-SC. **a** Results of biological process from GO analysis. Target genes of HRL-SC enriched in response to hypoxia and oxidation–reduction process. **b** The enrichment analysis performed with GSEA showed that HRL-SC enriched in hypoxia-related pathways. **c** Signaling pathways in KEGG enrichment analysis. **d** Expression analysis of target genes in pathways

To understand the function of key novel lncRNA, the target prediction of HRL-SC was performed, and the results showed that there was a total of 592 potential target genes identified from DEGs. The functions of target genes were analyzed through functional enrichment analysis. As shown in Fig. 5a, target genes of HRL-SC were significantly enriched in the response to hypoxia and the oxidation–reduction process. GSEA results confirmed this association that the differential expression of HRL-SC was associated with hypoxia conditions (Fig. 5b). In addition, pathway analysis indicated that PI3K/AKT signaling pathway, metabolic pathways and the other related pathway were identified (Fig. 5c). The expression analysis of target genes in pathways are shown in Fig. 5d. Among these, PDK1 was the most significantly differentially expressed target gene of HRL-SC in the PI3K/AKT signaling pathway (Fig. 5d). The details for prediction of target genes and functional annotation analysis are provided in Additional files 3, 4, 5.

### Knockdown of lncRNA HRL-SC inhibited the proliferation and migration of hDPSCs

**Fig. 6:**
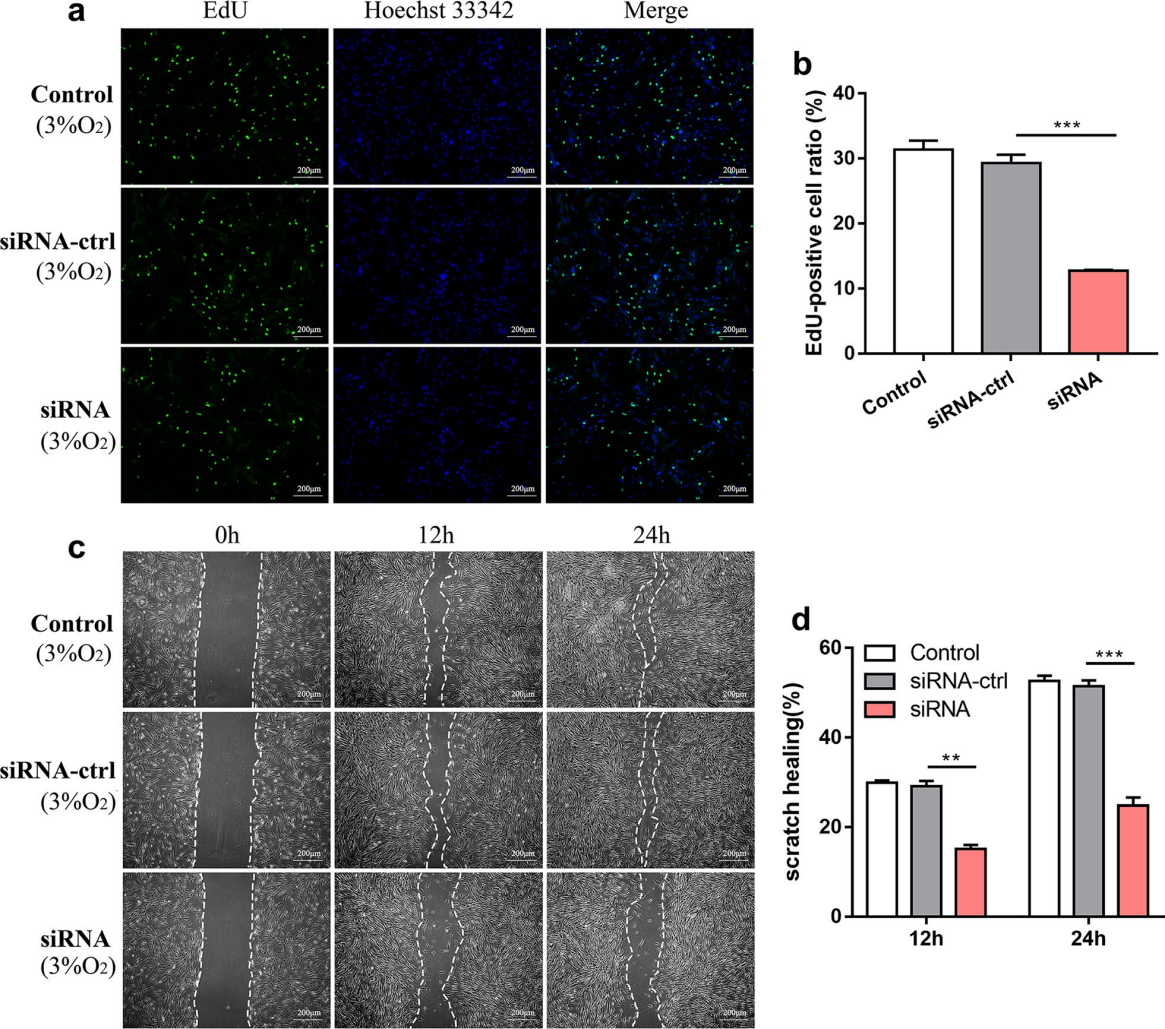
Knockdown of lncRNA HRL-SC inhibited the proliferation and migration of hDPSCs. **a** EdU assay of cell proliferation before and after knockdown of HRL-SC. EdU-positive cells were in red and cell nuclei were dyed in blue. **b** EdU-positive cell ratio in control group, siRNA-ctrl group and siRNA group. **c** Scratch wound healing assay was used to analyze migration capability before and after knockdown of HRL-SC. **d** Scratch wound healing area in control group, siRNA-ctrl group and siRNA group. **P < 0.01; ***P < 0.001

To confirm the role of HRL-SC in regulating proliferation and migration of hDPSCs, siRNA-mediated knockdown was performed and the expression levels of HRL-SC significantly decreased (Additional file 1: Fig. 1b). Moreover, the EdU assay showed that the cell proliferation rate in siRNA group was significantly lower than blank control and siRNA-ctrl group (Fig. 6a). Knockdown of HRL-SC inhibited proliferation of hDPSCs under hypoxia. Furthermore, the knockdown of HRL-SC also inhibited cell migration of hDPSCs. As shown in Fig. 6a, b lower migration rate was observed in siRNA group compared with blank control and siRNA-ctrl group.

### Knockdown of lncRNA HRL-SC inhibited the proliferation and migration of hDPSCs through the PI3K/AKT signaling pathway

**Fig. 7:**
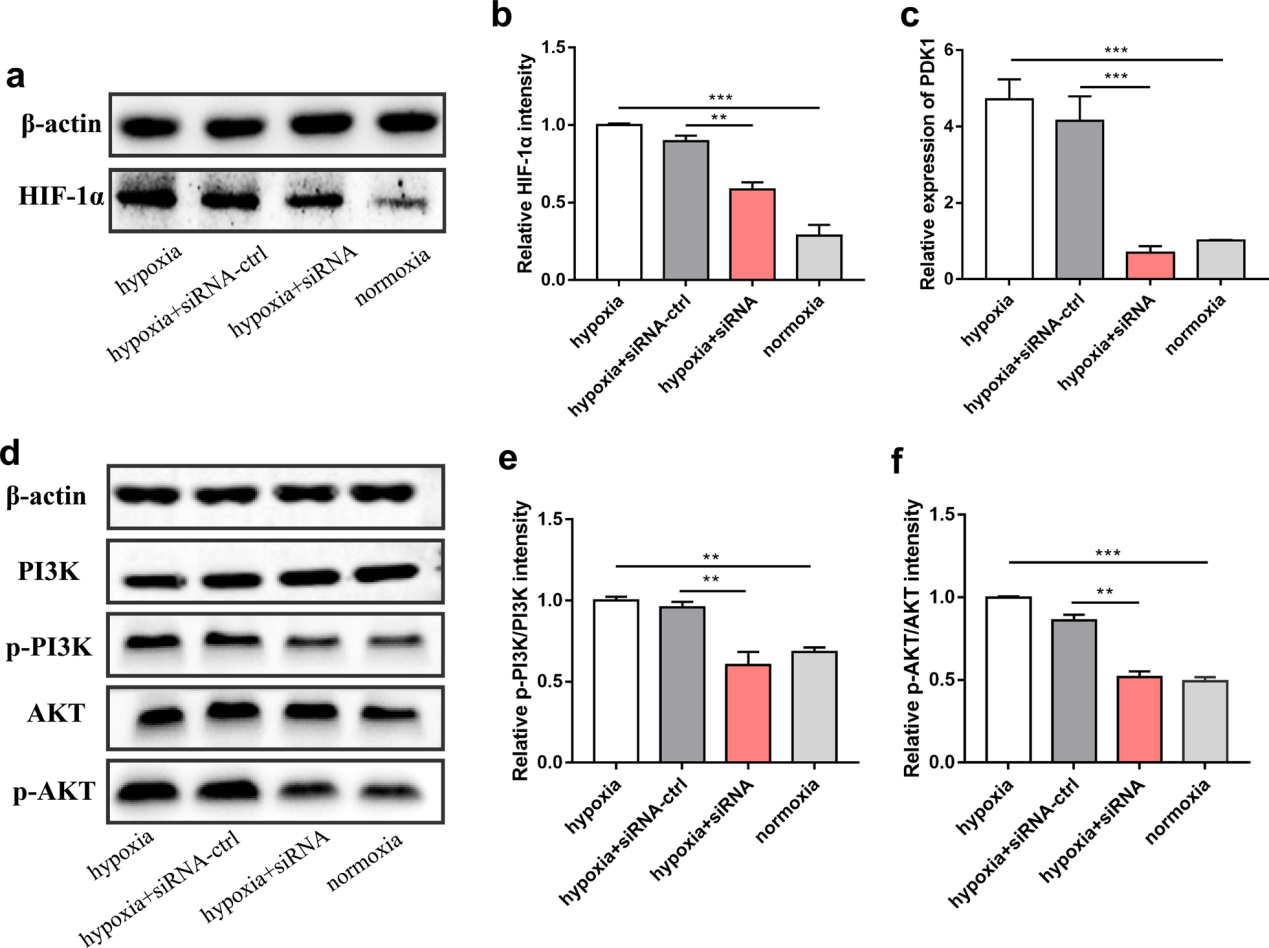
Knockdown of lncRNA HRL-SC downregulated the related proteins of the PI3K/AKT signaling pathway. **a**, **b** Western blot analysis showed that the expression level of HIF-1α decreased in the siRNA group. **c** qRT-PCR results showed that the expression levels of PDK1 decreased after knockdown of HRL-SC. **d** Western blot analysis showed that the expression level of p-AKT and p-PI3K decreased in the siRNA group. **e**, **f** Densities analysis of protein bands showed decreased ratios of p-AKT/AKT and p-PI3K/PI3K. **P < 0.01; ***P < 0.001

**Fig. 8:**
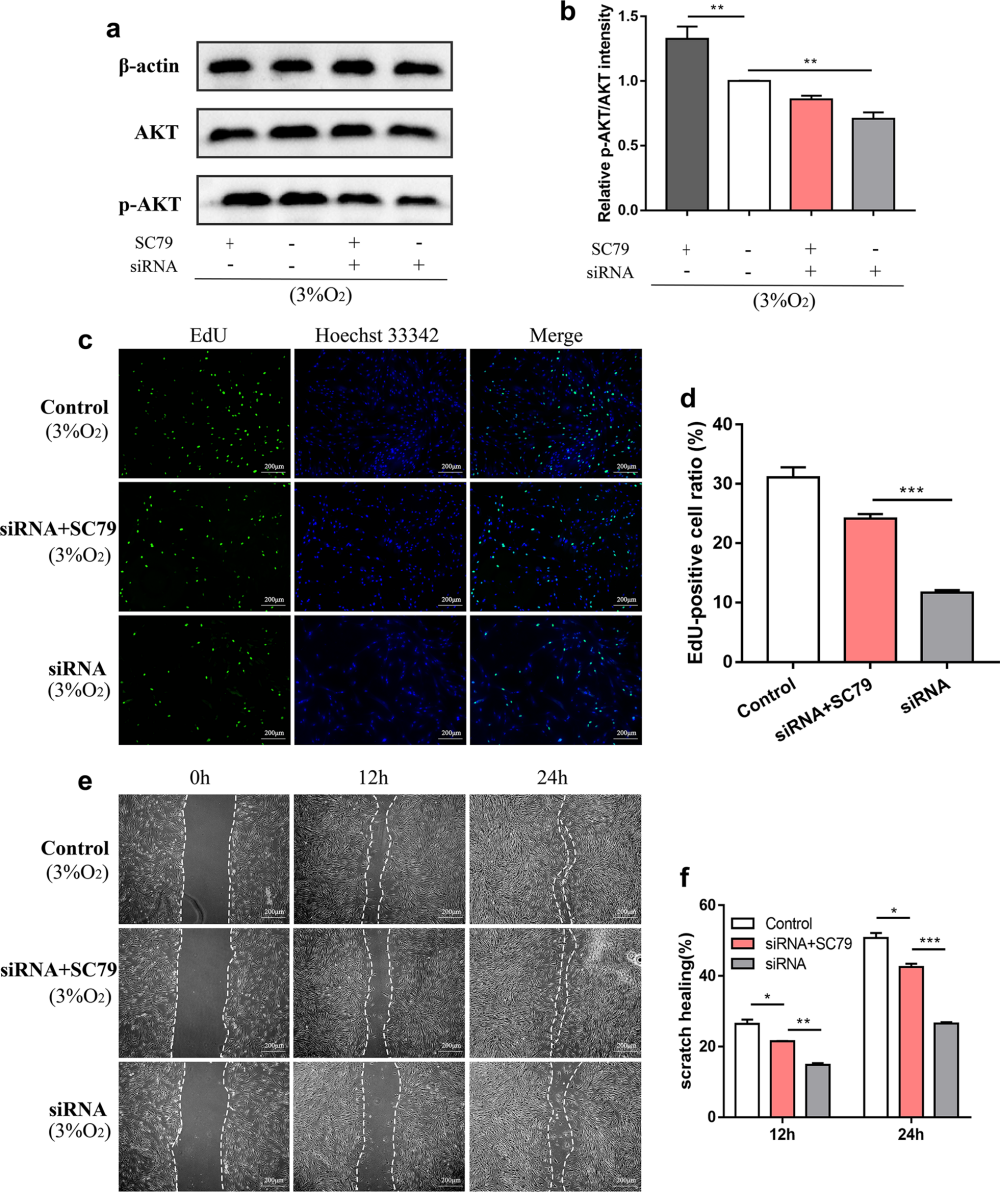
SC79 partially rescued the inhibitory effect of proliferation and migration caused by the knockdown of HRL-SC. **a**, **b** Western blot analysis showed that SC79 increased expression of p-AKT. **c** EdU assay of cell proliferation before and after addition of SC79. EdU-positive cells were in red and cell nuclei were dyed in blue. **d** EdU-positive cell ratio in control group, siRNA + SC79 group and siRNA group. **e** Scratch wound healing assay was performed to analyze migration capability before and after the addition of SC79. **f** Scratch wound healing area in control group, siRNA + SC79 group and siRNA group. *P < 0.05; **P < 0.01; ***P < 0.001

Our previous study demonstrated that PI3K/AKT signaling pathway-related marker proteins were increased in hDPSCs under hypoxia. Besides, the expression levels of PDK1, one of the potential target genes of HRL-SC, and HIF-1α increased during hypoxia exposure but decreased after the knockdown of HRL-SC (Fig. 7a–c). Combining with the results from pathway analysis of HRL-SC target genes, we speculated that HRL-SC may regulate proliferation and migration of hDPSCs through PI3K/AKT signaling pathway. To test this hypothesis, the expression levels of PI3K/AKT-related marker proteins were examined after the knockdown of HRL-SC. The results revealed that knockdown of HRL-SC decreased ratios of p-AKT/AKT and p-PI3K/PI3K, inhibiting the activation of the signaling pathway (Fig. 7d–f). Next, we added AKT activator SC79 into the medium when cell transfection was performed. The western bolt analysis showed that SC79 enhanced AKT phosphorylation and upregulated the expression of p-AKT (Fig. 8a, b). Furthermore, the EdU assay and scratch wound healing assay revealed that SC79 promoted the proliferation and migration of hDPSCs under hypoxia after knockdown of HRL-SC (Fig. 8c–f). In general, the results demonstrated that HRL-SC regulated the proliferation and migration of hDPSCs under hypoxia through the PI3K/AKT signaling pathway.

## Discussion

hDPSCs are typically isolated and cultured under normoxia, which differs from the stem cell microenvironment [33]. As hDPSCs proliferate during extended culture under normoxia, senescence-associated β-galactosidase levels increase, which is a premature senescence phenotype of stem cells [34, 35]. Accumulated injury caused by high oxygen tension can be damaging to stem cells [36]. Although there are some controversies related to the effect of hypoxia on hDPSCs, most studies have suggested that low oxygen tension promotes the proliferation and migration of hDPSCs [15, 16, 30]. The oxygen concentration has been set from 1 to 5% in the studies of hDPSCs in vitro under hypoxia, and evidence points to 3% O_2_ as the ideal concentration [15, 17, 30, 37], probably because the oxygen concentration of rat incisor pulp is approximately 3% [12]. Similar results were also observed in our study. When cultured under hypoxia, scratch wound healing and EdU assays revealed that hDPSCs exhibited higher levels of proliferation and migration than those cultured under normoxia.

Studies on HRL have mostly focused on tumor stem cells [38–40]. For example, the novel lncRNA KB-1980E6.3 was identified in breast cancer stem cells under hypoxia and was suggested to maintain stemness through the lncRNA KB-1980E6.3/IGF2BP1/c-Myc axis [40]. The underlying mechanism of HRL in hDPSCs, which is also promoted by hypoxia, remains unclear.

In the present study, we identified 496 unannotated novel lncRNAs from RNA-Seq data. By analyzing the exon number, chromosome distribution, FPKM values and coding ability, we found that the novel lncRNAs we identified conformed to the basic characteristics of lncRNAs. Based on the results of differential expression analysis and qRT-PCR, we screened the key novel lncRNAs that may play an important role in promoting the proliferation and migration of hDPSCs under hypoxia. Functional enrichment analysis revealed that HRL-SC was involved in the response to hypoxia and other hypoxia-related biological processes. The biological mechanisms of lncRNAs are associated with their subcellular localization [22]. Some lncRNAs distributed in the nucleus are generally involved in transcription or epigenetic regulation [41]. The other lncRNAs distributed in the cytoplasm are related to posttranscriptional regulation, including splicing, editing, transport, translation, degradation of mRNA and miRNA sponges [42]. The RNA FISH revealed that HRL-SC was located in the cell nucleus under normoxia but accumulated in the cell cytoplasm under hypoxia. The increased expression level and change in location of HRL-SC were observed from normoxia to hypoxia. We then determined the expression level of HRL-SC at different oxygen concentrations with different treatment times to observe the expression pattern of HRL-SC. Interestingly, dynamic expression levels of HRL-SC were observed in hDPSCs under hypoxia. Expression of HRL-SC was highest at 3% O_2,_ which is considered the ideal oxygen concentration for in vitro culture of hDPSCs. However, HRL-SC expression at 1% O_2_ was lower and similar to that in normoxia, probably because 1% O_2_ was not the ideal oxygen concentration for hDPSCs, and hypoxic injury that affects cell metabolism might occur under severe hypoxia [43]. Combined with the bioinformatic analysis, we demonstrated that HRL-SC functions as a hypoxia-responsive lncRNA to regulate hDPSCs.

To inhibit the functions of HRL-SC in hDPSCs under hypoxia, we used siRNA, an efficient silencing method for cytoplasmic lncRNA [44], to knockdown its expression. Knockdown of HRL-SC inhibited both the proliferation and migration of hDPSCs under hypoxia, but the negative control siRNA did not produce harmful effects. Physiological oxygen tension under hypoxic conditions is a necessary condition for the activation of hypoxia‐inducible factor-1α (HIF-1α) to maintain and protect stem cells [53, 54]. The energy supply of stem cells under hypoxia switches from oxidative phosphorylation to the glycolytic pathway, which is induced by HIF-1α [45]. It has also been reported that activation of AKT enhances HIF-1α translation. AKT is the core regulator of PI3K/AKT signaling, which is activated upon phosphorylation [46]. AKT phosphorylation is initiated by PI3K, which recruits AKT and PDK1 to the cell membrane, and PDK1 then phosphorylates AKT site Thr308 to partially activate AKT [47]. PI3K/AKT is constitutively activated in stem cells under hypoxia [48], and alteration of PI3K/AKT phosphorylation was also observed in our results. Some lncRNAs were demonstrated to regulate the activity of stem cells via the PI3K/AKT signaling pathway. For example, it was observed that lncRNA AK015322 promotes the proliferation of hair follicle stem cells via the PI3K/AKT signaling pathway [49]. The decreased expression of p-AKT and p-PI3K showed that HRL-SC might initiate constitutive activation of PI3K/AKT signaling in a variety of ways. Similar to our study, knockdown of lnc-ORA, a novel lncRNA that was identified from RNA-Seq data, reduced the expression of p-AKT and p-PI3K in adipose tissue, and lnc-ORA was considered to regulate adipocyte differentiation via the PI3K/AKT signaling pathway [50]. Because PDK1 was one of the potential target genes of HRL-SC and its expression decreased after knockdown of HRL-SC, one possibility is that HRL-SC modulated PDK1 to activate the PI3K/AKT signaling pathway [51].

For further validation, we added SC79 to the medium after knockdown of HRL-SC. SC79 is an AKT activator that induces the phosphorylation of AKT to activate the PI3K/AKT signaling pathway [52]. The activation of p-AKT by SC79 was confirmed. As the results showed, SC79 partially rescued the inhibitory effect of HRL-SC knockdown on proliferation and migration in hDPSCs under hypoxia. Therefore, we considered that the novel HRL-SC promotes the proliferation and migration of hDPSCs under hypoxia via the PI3K/AKT signaling pathway.

There are still several potential limitations. For example, the binding sites and target genes of HRL-SC still need plenty of experiments to confirm. In addition, there is no perfect cell model with the ability to completely mimic all characteristics of the hDPSCs in human dental pulp, so the experiment results may not be entirely consistent with the actual situation. The specific regulatory mechanism will be investigated in our future studies.

## Conclusions

In conclusion, we identified a novel HRL-SC from RNA-Seq data of hDPSCs under hypoxia compared with normoxia. HRL-SC is a hypoxia-responsive lncRNA that promotes the proliferation and migration of hDPSCs through the PI3K/AKT signaling pathway. These findings contribute to an understanding of the mechanism of lncRNA in hDPSCs under hypoxia and to the application of hDPSCs in pulp regeneration.

## Supplementary Information

**Additional file 1: Fig. S1.** The sequence information of lncRNA HRL-SC and validation of siRNA-mediated knockdown using qRT-PCR.

**Additional file 2.** Details of differentially expressed mRNAs, known lncRNAs and novel lncRNAs under hypoxia compared with normoxia.

**Additional file 3.** Details of target genes of HRL-SC.

**Additional file 4.** Details of GO analysis for HRL-SC.

**Additional file 5.** Details of KEGG pathway analysis for HRL-SC.

## Data Availability

Not applicable.

## Abbreviations

HRL: Hypoxia-responsive lncRNA
CNCI: Coding-Non-Coding-Index
CPAT: Coding Potential Assessment Tool
CPC: Coding Potential Calculator
DAVID: Database for Annotation Visualization and Integrated Discovery
DEGs: Differentially expressed genes
DENLs: Differentially expressed novel lncRNAs
DMEM: Dulbecco modified eagle’s medium
FBS: Fetal bovine serum
FISH: Fluorescence in situ hybridization
GO: Gene ontology
GSEA: Gene set enrichment analysis
hDPSCs: Human dental pulp stem cells
HIF: Hypoxia inducible factor
KEGG: Kyoto Encyclopedia of Genes and Genomes
lncRNAs: Long noncoding RNAs
Oct-4: Octamer-binding protein 4
PVDF: Polyvinylidene difluoride
siRNA: Small interfering RNA
SOX2: SRY-Box transcription factor 2
