# Supplementary material for: A novel hypoxic lncRNA, HRL-SC, promotes the proliferation and migration of human dental pulp stem cells through the PI3K/AKT signaling pathway
Source: Stem Cell Res Ther. 2022 Jun 28;13:286. doi: 10.1186/s13287-022-02970-5 (PMC9241257; doi:10.1186/s13287-022-02970-5)
Supplement: Supplementary file 1 — Additional file 1: Fig. S1. The sequence information of lncRNA HRL-SC and validation of siRNA-mediated knockdown using qRT-PCR. [file 13287_2022_2970_MOESM1_ESM.docx]

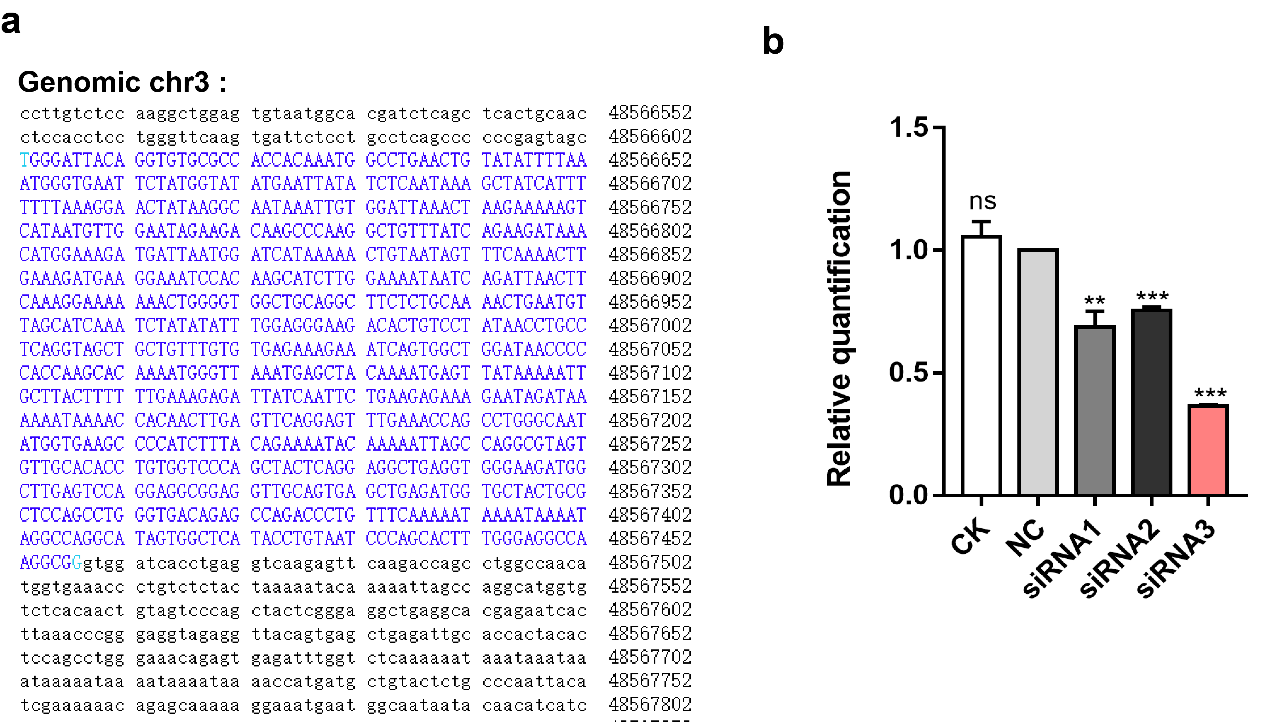


Supplementary Fig.1 **a** The sequence information of lncRNA HRL-SC. **b** Validation of siRNA-mediated knockdown using qRT-PCR. The transfection of siRNA3 could obviously decreased the expression level of HRL-SC.
